# Norepinephrine titration in patients with sepsis-induced encephalopathy: cerebral pulsatility index compared to mean arterial pressure guided protocol: randomized controlled trial

**DOI:** 10.1186/s12871-024-02814-0

**Published:** 2025-01-04

**Authors:** Mai Salah Salem, Motaz Amr Abosabaa, Mohamed Samir Abd El Ghafar, Hala Mohey EI-Deen Mohamed EI-Gendy, Salah El-din Ibrahim Alsherif

**Affiliations:** Department of Anesthesia, Surgical Intensive Care and Pain Medicine, Faculty of Medicine, Tanta University Hospitals, Tanta, Gharbya Egypt

**Keywords:** Sepsis-induced encephalopathy, TCD Pulsatility index, Mean arterial pressure, Norepinephrine titration, Cerebral hypoperfusion, ICU mortality

## Abstract

**Background:**

Although surviving sepsis campaign (SSC) guidelines are the standard for sepsis and septic shock management, outcomes are still unfavourable. Given that perfusion pressure in sepsis is heterogeneous among patients and within the same patient; we evaluated the impact of individualized hemodynamic management via the transcranial Doppler (TCD) pulsatility index (PI) on mortality and outcomes among sepsis-induced encephalopathy (SIE) patients.

**Methods:**

In this prospective, single-center randomized controlled study, 112 patients with SIE were randomly assigned. Mean arterial pressure (MAP) and norepinephrine (NE) titration were guided via the TCD pulsatility index to achieve a pulsatility index < 1.3 in Grou*p* I, whereas the SSC guidelines were used in Grou*p* II to achieve a MA*P* ≥ 65 mmHg. The primary outcome was intensive care unit (ICU) mortality and the secondary outcomes were; MA*P* that was measured invasively and values were recorded; daily in the morning, at the end of NE infusion and the end of ICU stay, duration of ICU stay, cerebral perfusion pressure (CPP), sequential organ failure assessment (SOFA) score, norepinephrine titration and Glasgow coma scale (GCS) score at discharge.

**Results:**

ICU mortality percentage wasn`t significantly different between the two groups (*p* value 0.174). There was a significant increase in the MA*P* at the end of norepinephrine infusion (mean value of 69.54 ± 10.42 and *p* value 0.002) and in the GCS score at ICU discharge (Median value of 15 and *p* value 0.014) in the TCD group, and episodes of cerebral hypoperfusion with CP*P* < 60 mmHg, were significantly lower in the TCD grou*p* (median value of 2 and *p* value 0.018). Heart rate values, number of episodes of tachycardia or bradycardia, Total norepinephrine dosing, duration of norepinephrine infusion, SOFA score, serum lactate levels, and ICU stay duration weren`t significantly different between the two groups.

**Conclusions:**

Individualizing hemodynamic management via the TCD pulsatility index in SIE patients was not associated with significant mortality reduction. However, it reduces episodes of cerebral hypoperfusion and improves GCS outcome but doesn’t significantly affect heart rate values, SOFA score, serum lactate level, length of ICU stay, total NE dosing, and duration of NE infusion.

**Trial registration:**

The clinical trial was registered on clinucaltrials.gov under the identifier NCT05842616 https://clinicaltrials.gov/study/NCT05842616?cond=NCT05842616&rank=1 on 6-May-2023 before the enrolment of the first patient.

**Supplementary Information:**

The online version contains supplementary material available at 10.1186/s12871-024-02814-0.

## Background

Sepsis-induced encephalopathy (SIE) is a serious complication with a high mortality rate [1]. Alterations in cerebral auto-regulatory mechanism together with microcirculatory affection could explain local cerebral hypoperfusion even without severe systemic hypotension [2]. Restoring sufficient organ perfusion is crucial for surviving sepsis, and preserving adequate cerebral perfusion may improve outcomes in patients with SIE [3].

Alteration in cerebral autoregulation together with microcirculatory alterations could explain local hypoperfusion even without severe systemic hypotension [3]. Autoregulation maintains constant perfusion to vital organs including the brain, kidney, and heart within MA*P *between 60 to 150 mmHg [4]. Cerebral-autoregulation maintains constant cerebral perfusion despite fluctuating MA*P* through adjustment of cerebral blood vessels diameters (cerebrovascular reactivity). MA*P* limits of functional cerebral autoregulation vary among individuals, and cerebral blood flow (CBF) is passive to the changes in MA*P*outside these limits [3].

The MAP, in fact, is not organ perfusion pressure; perfusion pressure is heterogeneous, not only among patients but also within the same patient during the progression of septic shock [5]. This discrepancy is what makes hemodynamic management in SIE, especially challenging and requires close, bedside monitoring and vasopressor titration [5]. To date, it is not clear whether the SSC guidelines for MA*P *target ≥ 65 mmHg are reasonable for maintaining adequate cerebral perfusion pressure in SIE patients [6]. TCD is considered an addition to conventional diagnostic methods to optimize the hemodynamic management of SIE patients by targeting systemic blood pressure, which is sufficient to maintain adequate peripheral organ perfusion and reduce the influence on brain homeostasis [6]. The elevation of the TCD pulsatility index is associated with elevated cerebrovascular resistance and correlated with a greater occurrence of delirium [7]. A pulsatility index value > 1.3 may be indicative of brain dysfunction in sepsis [8] The utilization of noninvasive surrogates for cerebral perfusion and cerebral vasoreactivity, such as the TCD pulsatility index, allows personalized estimation of optimal cerebral perfusion pressure (CPP) in SIE patients and is associated with better outcomes [7].

The current clinical trial aims to compare the ICU mortality and other secondary outcomes between the TCD grou*p* and the MA*P* group.

## Methods

### Inclusion criteria

Patients aged 18 years or older diagnosed with sepsis-induced encephalopathy with septic shock were included.

- ➢ Sepsis was defined as suspected or evident infection and patients who had two or more quick SOFA scores: [9]
  - Altered mentation.
  - Respiratory rate ≥ 22 breaths per minute.
  - Systolic blood pressure ≤ 100 mmHg.
- ➢ Septic shock was defined as sepsis-induced hypotension that persists despite adequate fluid resuscitation and serum lactate level ≥ 2 mmol/l [10].
- ➢ Encephalopathy was diagnosed as GCS less than 15.

### Exclusion criteria

Patient refusal, cerebral infection or known cerebral lesion, severe internal carotid artery (ICA) stenosis > 70%, pregnancy, drug intoxication, and patients supported with an intra-aortic balloon pump.

### Study design

**Fig. 1:**
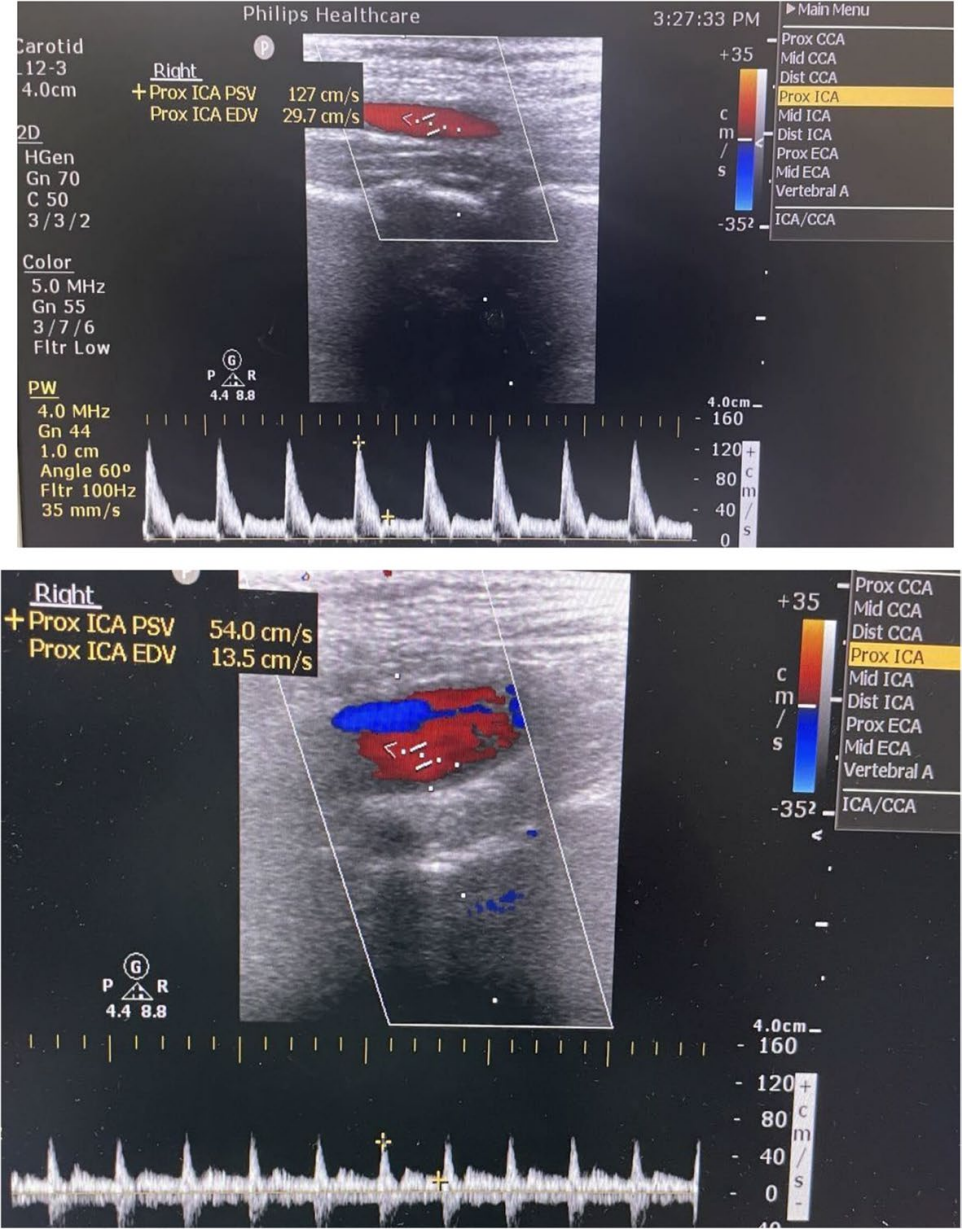
Ultrasound assessment for diagnosis of ICA stenosis showing normal ICA

Patients who met these criteria; were enrolled in this trial and were randomly and equally divided into two groups; Ultrasound assessment of the ICA and common carotid artery. (CCA) was performed to exclude cases with severe carotid artery stenosis > 70%. The criteria for diagnosis of severe ICA stenosis (> 70%) include the following: [11] PSV of ICA ≥ 215 cm/s, EDV of ICA ≥ 65 cm/s, internal to common carotid artery PSV ratio ≥ 3.7, as shown in Fig. 1.

All patients were managed according to the SSC guidelines for managing septic shock [12] which started within the first hour except for norepinephrine (NE) titration guided by the TCD pulsatility index in grou*p* I and the MA*P* in grou*p* II. Fluid resuscitation was started by administrating lactated ringer solution at a rate of 4 to 6 ml/kg.

Re-evaluation was performed after 15 min. If the MA*P* was still ≤ 65 mmHg, we continued resuscitation at a rate of 4 to 6 ml/kg. Re-evaluation was also performed after 15 min; until 30 ml/kg was reached. Norepinephrine was started when the patient was still hypotensive with a MAP < 65 mmHg, either during or after fluid resuscitation, even peripherally, to avoid delay until central venous access was secured. NE was the vasopressor used in this study. The norepinephrine formula used in this clinical trial was NE tartrate (ampoule preparation contains 8 mg NE tartrate, equivalent to 4 mg NE base). For patients with inadequate MAP, despite NE dose escalation u*p* to 0.5 µg/kg/min, we added epinephrine as vasopressin wasn`t commercially available.

At the same time, the following steps were done; serum lactate measurements, obtaining appropriate routine microbiologic culture, broad-spectrum antibiotic administration, and rapid source control.

In grou*p* I, patients were placed in a supine position with the head of the bed elevated to approximately 30 to 45 degrees. Three measurements were taken, and the average value was recorded. The flow velocity of the middle cerebral arteries was measured using a low frequency 1–5 MHZ (phased-array) transcranial Doppler probe on both sides. The probe was positioned over the temporal bone window above the zygomatic arch just anterior to the patient’s ear at the level of the eye, and then a sliding motion was performed to scan through the adjacent brain tissue.

First, the temporal bone was identified, followed by the midbrain, which is butterfly-shaped, was identified. Just anterior to the midbrain, the circle of Willis was located and identified using color-coded sonography.

The middle cerebral artery (MCA) was identified as a linear, red structure as the blood flow was directed toward the ultrasound transducer. The blood flow velocity within the vessel was measured via pulsed wave (PW) Doppler.

The normal flow velocity of the MCA was associated with a stee*p* upstroke in systole and stepwise deceleration in diastole.

**Fig. 2:**
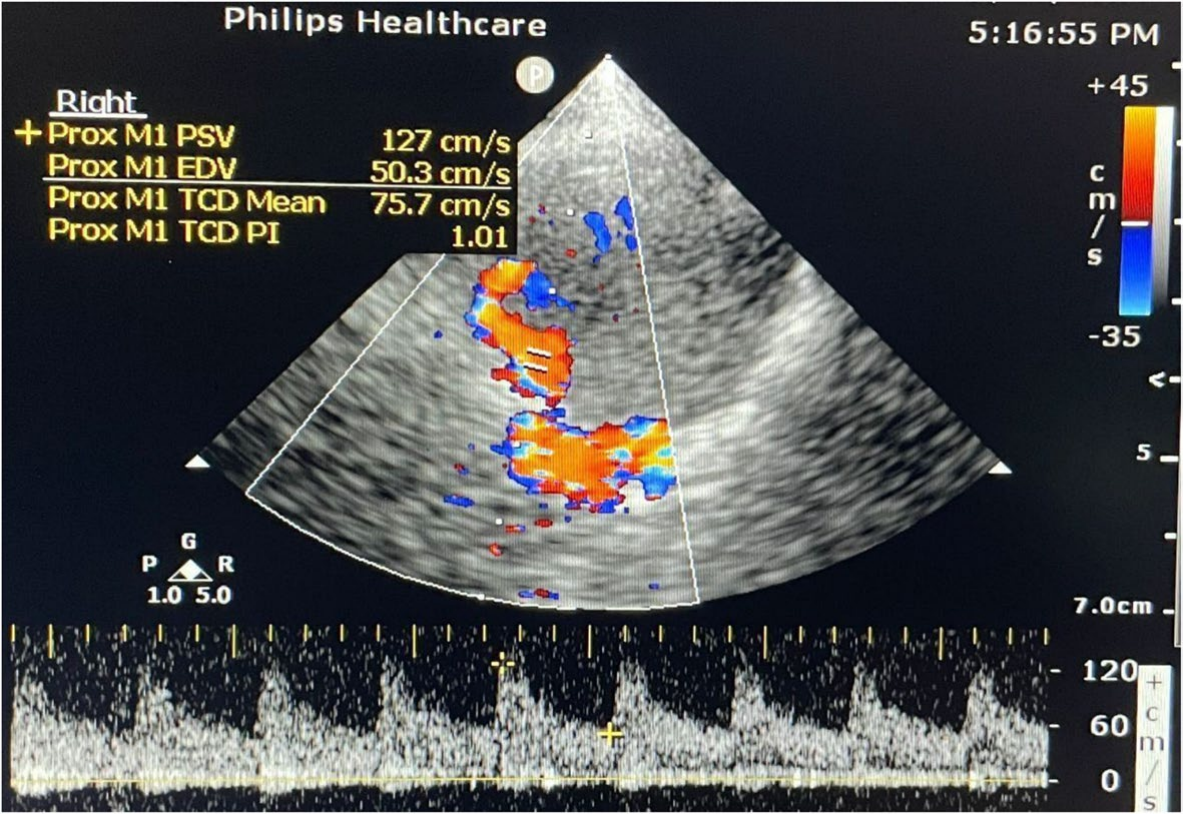
Measurements of pulsatility index of right MCA (PSV 127 cm/s, EDV 50.3 cm/s, MFV 75.7 cm/s, PI 1.01)

The peak systolic velocity (PSV), end-diastolic velocity (EDV), and mean flow velocity (MFV) were measured. The pulsatility index (PI) was calculated by using the following equation: {PI = (PSV_ EDV)/ (MFV) [13]}, as shown in Fig. 2.

Our goal was to guide norepinephrine titration to maintain a pulsatility index below 1.3. The pulsatility index was assessed twice daily unless hypotension occurred; reassessment was performed at this time to guide NE titration.

CP*P* was calculated by TCD in both groups at the time of hypotensive episodes via the following equation: CP*P* = MAP x (EDV/MFV) + 14. [14] 14 mmHg is a calibration (zeroing) parameter established. Its normal range is 60–80 mm Hg [15].

In grou*p* II, the MA*P* was measured continuously, and our goal was to guide norepinephrine titration to maintain MA*P* ≥ 65 mmHg.

ICU mortality was the primary outcome, whereas MAP, CPP, norepinephrine titration, SOFA score, ICU stay duration, and GCS values were the secondary outcomes.

### Data collection

All registered patients were subjected to the following measurements:

The demographic data, hemodynamic parameters (MAP, heart rate), serum lactate level, SOFA score at admission and discharge, total norepinephrine dosing, duration of norepinephrine infusion, CPP, encephalopathy outcome according to GCS score, duration of ICU stay and ICU mortality.

### Sample size analysis

The sample size and power analysis were calculated using the Epi-Info software statistical package created by the World Health Organization and Center for Disease Control and.

Prevention, Atlanta, Georgia, USA version 2002.

The criteria used for sample size calculation were as follows:

−95% confidence limit.
−80% power of the study.

The expected incidence of mortality cases of sepsis-induced encephalopathy in the best intervention grou*p* is 20% compared to 45% in the least favorable intervention group.

The sample size based on the previous criteria was found at *N* = 56 in each group.

### Statistical analysis

Statistical analysis was done by SPSS v26 (IBM Inc., Chicago, IL, USA). Shapiro-Wilks test and histograms were used to evaluate the normality of the distribution of data. Quantitative parametric variables were presented as mean and standard deviation (SD) and compared between the two groups utilizing an unpaired Student’s t-test. Quantitative non-parametric data were presented as the median and interquartile range (IQR) and were analyzed by the Mann Whitney-test. Qualitative variables were presented as frequency and percentage (%) and analyzed utilizing the Chi-square test or Fisher’s exact test when appropriate. A two-tailed *P* value < 0.05 was considered statistically significant.

## Results

### Randomization

The patients were randomly assigned into two equal comparable groups using computer-generated random numbers, with sequentially numbered closed opaque envelopes containing either the intervention grou*p* (Grou*p* I) or the control grou*p* (Grou*p* II).

**Fig. 3:**
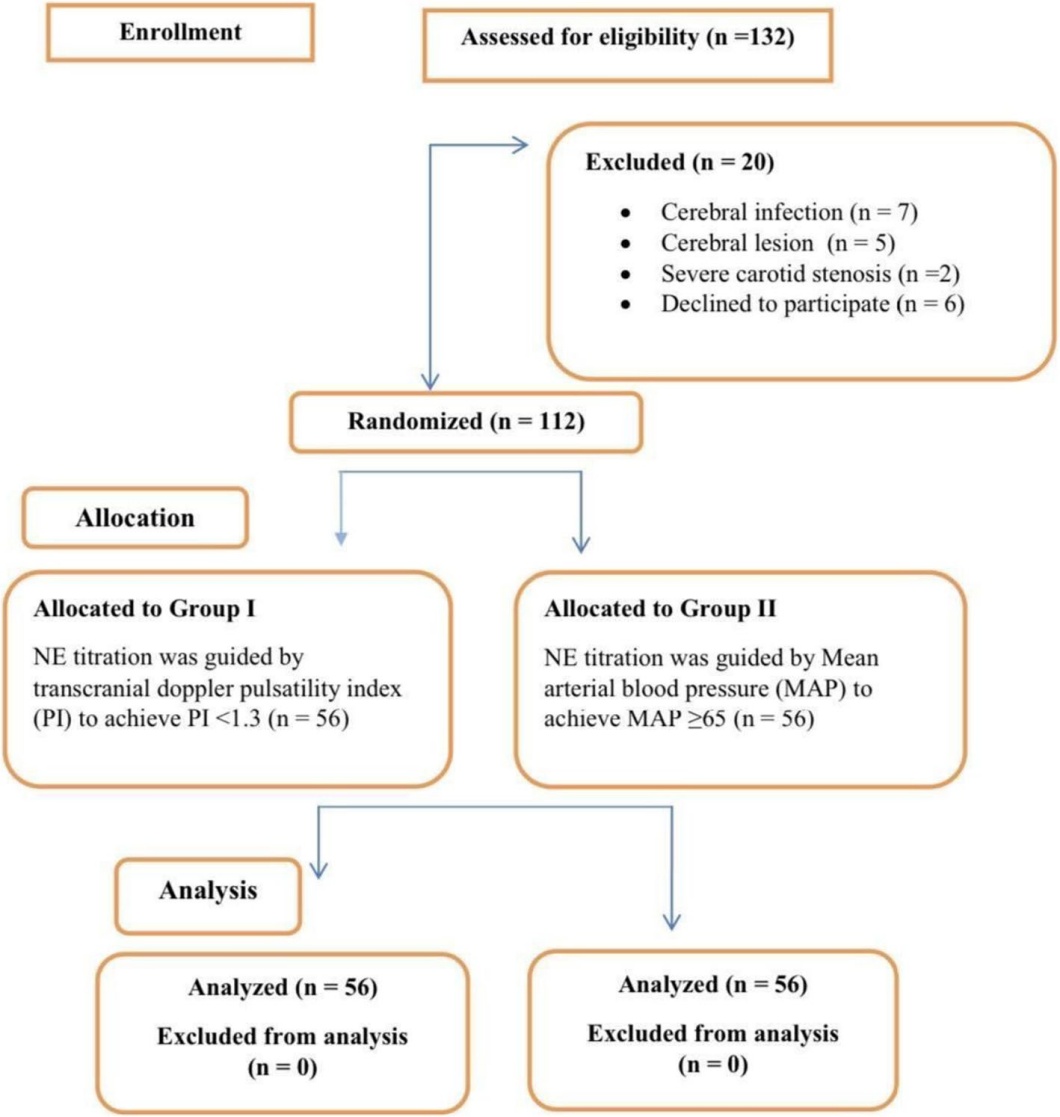
Patient flowchart summarizing enrolment, allocation, follow-up, and analysis in the study protocol

In our clinical trial, 132 patients were assessed for eligibility, and 20 patients were excluded (7 with cerebral infection, 5 with cerebral lesion, 2 with severe carotid stenosis, and 6 cases declined to participate), as shown in Fig. 3. All patients who met the inclusion criteria were randomly and equally divided into two groups, with 56 patients in each group. Analysis was performed, and no patients were excluded from the analysis.

According to the demographic data (age, sex, BMI and etiology of septic shock), no significant difference was found between the two groups as shown in Table 1.

**Table 1:** Patient characteristics

Variables	TCD group	MA*P* group	Test of significance
**Sex**			χ^2^ = 0.036
Male	24 (42.9%)	25 (44.6%)	
Female	32 (57.1%)	31 (55.4%)	
**Age (years)**			T 0.705
Mean ± SD	52.27 ± 13.87	54.13 ± 14.01	
**BMI (kg/m**^**2**^**)**			T 0.575
Mean ± SD	25.96 ± 3.31	25.59 ± 3.61	
**Etiology of septic shock**			χ^2^ = 0.511
** Pneumonia**	19 (33.9%)	16 (28.5%)	
** UTI**	14 (25%)	14 (25%)	
** Post operative sepsis**	14 (25%)	16 (28.5%)	
** Intra-abdominal sepsis**	9 (16%)	10 (17.8%)	

*T* Student t-test, χ^2^ Chi square test, *SD *Standard deviation

MA*P* values showed a significant increase in the TCD grou*p* at the end of NE infusion and at the end of ICU stay (*p* value = 0.002 and 0.007 respectively). However, no significant difference was recorded in the number of episodes of hypotension (MA*P* < 65 mmHg) between the two groups. There were no significant differences in heart rate values, episodes of tachycardia or episodes of bradycardia during NE infusion between the two groups. In terms of CPP, the number of episodes of cerebral hypoperfusion with CP*P* < 60 mmHg, was significantly higher in the MA*P* grou*p* (*p* value = 0.018) as shown in Table 2.

**Table 2:** Hemodynamic parameters during norepinephrine infusion

Variables	TCD grou*p*	MA*P* grou*p*	Test of significance	*P* value
**MA*P* (mmHg)**				
**Baseline**				
Mean ± SD	52.86 ± 5.99	52.66 ± 4.66	T 0.194	**0.847**
**End of NE infusion**				
Mean ± SD	69.54 ± 10.42	63.96 ± 7.99	T 3.174	**0.002**
**End of ICU stay**				
Mean ± SD	70.30 ± 10.64	65.18 ± 9.09	T 2.740	**0.007**
**Frequency of hypotensive episodes during NE infusion**				
Median (IQR)	8.0 (5.50 – 21.0)	7.0 (4.50 – 23.0)	U 1526.50	**0.809**
**HR during NE infusion (beat/min)**				
**Baseline**				
Mean ± SD	95.13 ± 15.92	98.05 ± 12.22	T 1.092	**0.277**
**End of NE infusion**				
Mean ± SD	84.34 ± 16.28	85.30 ± 16.51	T 1.092	**0.756**
**End of ICU stay**				
Mean ± SD	86.18 ± 14.89	86.50 ± 15.84	T 1.092	**0.912**
**Frequency of episodes of tachycardia**				
Median (IQR)	6.0 (4.50 – 10.50)	8.0 (6.0 – 10.0)	U 1282.00	**0.095**
**Frequency of episodes of bradycardia**				
Median (IQR)	1.0 (1.0 – 4.0)	2.0 (1.0 – 6.0)	U 1443.00	**0.456**
**CP*P* during hypotensive episodes (mmHg)**				
**Baseline**				
Median (IQR)	58.0 (52.0 – 60.0)	56.0 (53.0 – 58.50)	U 1548.00	**0.906**
**End of NE infusion**				
Median (IQR)	60.0 (52.50 – 62.0)	60.0 (48.0 – 63.0)	U 1524.00	**0.797**
**Frequency of episodes of cerebral hypoperfusion**				
Median (IQR)	2.0 (1.0 – 4.0)	3.0 (2.0 – 7.0)	U 1168.00	0.018^*^

*T* Student t-test, *U* Mann Whitney test, *SD* Standard deviation, *IQR* Inter quartile range, *p* *p* value for comparing between the two studied groups

^*^Statistically significant at *p* ≤ 0.05

Glasgow coma scale values were significantly higher in the TCD grou*p* at the ICU discharge (*p* value = 0.014). There were no significant differences in the serum lactate level, SOFA score, ICU stay length, and ICU mortality between the groups as shown in Table 3.

**Table 3:** Clinical outcomes

Variables	TCD grou*p*	MA*P* grou*p*	Test of significance	*P* value
**Serum lactate (mmol/L)**				
**Baseline**				
Median (IQR)	3.80 (3.37 – 4.22)	3.80 (3.45 – 4.20)	U 1545.50	**0.896**
**End of NE infusion**				
Median (IQR)	2.50 (2.14 – 5.80)	2.65 (2.20 – 5.90)	U 1437.00	**0.445**
**End of ICU stay**				
Median (IQR)	1.98 (1.84 – 5.80)	2.0 (1.86 – 5.90)	U 1549.50	**0.914**
**SOFA score**				
**Baseline**				
Median (IQR)	4.0 (3.0 – 7.0)	5.0 (3.0 – 8.0)	U 1378.50	**0.260**
**End of ICU stay**				
Median (IQR)	2.0 (1.0 – 11.0)	2.0 (1.0 – 11.50)	U 1362.50	**0.225**
**GCS**				
**Baseline on ICU admission**				
Median (IQR)	11.0 (10.0 –12.0)	11.0 (9.0 – 12.0)	U 1392.50	**0.301**
**Outcome at ICU discharge**				
Median (IQR)	15.0 (8.0 –15.0)	14.0 (5.0 – 15.0)	U 1169.50	0.014^*^
**ICU stay (days)**				
Median (IQR)	6.0 (5.0 – 7.0)	6.0 (5.0 – 7.0)	U 1419.50	**0.379**
**ICU mortality**				
Percentage	32.1	44.6	χ^2^ 1.850	**0.174**
**Total NE dosing (mg)**				
Median (IQR)	94.45 (65.85_248.0)	113.5 (60.05_294.0)	U 1384.00	**0.284**
**Duration of NE infusion (days)**				
Median (IQR)	4.0 (3.0 – 7.0)	5.0 (3.0 – 7.0)	U 1382.50	**0.275**

*U* Mann Whitney test, χ^2^ Chi square test *IQR* Inter quartile range, *p* *p* value for comparing between the two studied groups

*Statistically significant at *p*≤ 0.05

There were no significant differences in total NE dosing or duration of NE infusion between the two groups as shown in Table 3.

**Fig. 4:**
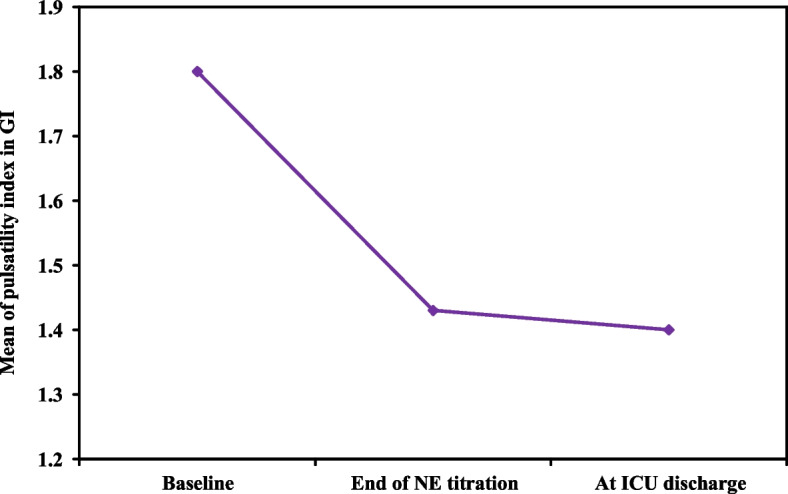
Pulsatility index measurements in TCD grou*p*

Compared with the baseline values, the TCD pulsatility index values in Grou*p* I were significantly lower at the end of NE infusion (*p* value < 0.001) and ICU stay (*p* value < 0.001) as shown in Table 4 and Fig. 4.

**Table 4:** Descriptive analysis of pulsatility index values in grou*p* I (TCD Pulsatility index guided MA*P* and NE titration)

Pulsatility index	TCD grou*p*	P0
**Baseline**		
Median (IQR)	1.60 (1.31 – 2.15)	
**End of NE infusion**		
Median (IQR)	1.20 (1.15 – 1.89)	< 0.001*
**End of ICU stay**		
Median (IQR)	1.14 (1.10 – 1.89)	< 0.001*

*IQR* Inter quartile range, *SD* Standard deviation, p_0_: *p* value for Post Hoc Test (Dunn’s) for Friedman test for comparing between baseline values in the first day and each other periods

^*^Statistically significant at *p* ≤ 0.05

## Discussion

Cerebral disorders of microcirculation and cerebral perfusion insufficiency are implicated in the pathogenesis of sepsis-induced encephalopathy [16]. The conventional MA*P* target of the current SSC guidelines (MA*P*≥ 65 mmHg) may not be suitable for all patients, especially hypertensive patients, and this may lead to cerebral perfusion insufficiency [16]. Patients in septic shock have impaired cerebral autoregulation, that is related to the occurrence of sepsis-induced encephalopathy [17]. Therefore, optimal hemodynamic management to ensure adequate cerebral perfusion may improve the prognosis of patients with SIE [17].

This study demonstrated that the TCD pulsatility index is an effective tool for individualizing hemodynamic management and guiding vasopressor titration to maintain adequate perfusion and minimize neurological complications of SIE. In the present study, the TCD pulsatility index was used to personalize hemodynamics during SIE management by titrating norepinephrine (NE) infusion to maintain a pulsatility index of less than 1.3 in grou*p* I.

A prospective observational study, conducted by Charalampos Pierrakos et al., (2014) [18] on 21 SIE patients, revealed a pulsatility index cut off value of 1.3 with 95% sensitivity and 88% specificity in SIE prediction and that it can be used in clinical practice to guide the resuscitation and monitoring of SIE patients, which is in line with our clinical trial target.

Additionally, a prospective randomized controlled study was performed by C Ben Miled et al., (2022) [19] on 50 patients with SIE on NE titration at the early stage of septic shock. They compared personalized TCD-guided hemodynamic management with the SSC recommendations using a pulsatility index cut off value of 1.3 to guide NE titration in the experimental group. In the current clinical trial, there was a significant decrease in the pulsatility index values at the end of NE infusion and ICU stay in grou*p* I, which was in agreement with the findings of the study conducted by C Ben Miled et al., (2022) [19], which reported a significant reduction in the pulsatility index values in the TCD grou*p* (*p* < 10^−4^).

In the current clinical trial, TCD PI-guided management of MA*P* in patients with SIE did not reduce ICU mortality (32.1%% vs. 44.6%%, *P* = 0.174). However, MA*P* values were significantly higher at the end of NE infusion (*p* value 0.002) and at the end of ICU stay (*p* value 0.007) in the TCD group. These results came in line with those of C Ben Miled et al., (2022) [19], who reported a significant increase in MA*P* values at the end of NE titration in the TCD grou*p* but was also associated with significant mortality reduction in the experimental group, in contrast to our clinical trial results. Additionally, in the study conducted by Qianyi Peng et al., (2024) [20] involving 51 patients with septic shock, 26 patients in the experimental grou*p* were resuscitated by using cerebral autoregulation-guided optimal MAP, at which the cerebrovascular reactivity was at its best and the tissue oxygen reactivity index was lowest, whereas, in the control group, MA*P* management was performed according to SSC guidelines. They reported that higher values of optimal MA*P* (mean value of 84.5 ± 12.2) were required in the experimental grou*p* than in the control grou*p* (mean value of 77.4 ± 11.8). Also HongyanPeng et al., (2024) [21] retrospectively collected data from 3,816 patients with sepsis-induced encephalopathy, at ICU admission and concluded that in case of MA*P* < 81.5 mmHg, an increase in MA*P* value was associated with a decreased risk of 28 day and in-hospital mortality (*P* < 0.05). An increase in MA*P* of about 5 mmHg was associated with a 15% reduction in 28-day mortality risk and a 14% reduction in in-hospital mortality risk, while for MAP ≥ 81.5 mmHg, there was no significant association between MA*P* and mortality risk (*P* > 0.05). In contrast to our results, the prospective multicentric cohort study performed by Lina Zhao et al., (2022) [22] involving 5861 patients in the SIE group, on vasopressors and 3172 patients in the non-SIE grou*p* revealed that a MA*P* ≥ 65 mmHg was associated with the lowest SIE incidence. These findings demonstrate that a MA*P* ≥ 65 mmHg is associated with adequate cerebral perfusion, consistent with the current SSC guidelines for MA*P* target.

In our clinical trial, there was no significant difference in episodes of tachycardia or bradycardia between the two groups. In accordance with our clinical trial, C Ben Miled et al., (2022) [19] reported that heart rate values recorded during NE titration were comparable between the two groups. Additionally, in this clinical trial, there was a significant reduction in the number of episodes of cerebral hypoperfusion in the TCD grou*p* (*p* value 0.018) and a significant increase in the GCS score at ICU discharge in the TCD grou*p* (*p* value 0.014) compared with those in the MA*P* group. This finding was also in line with that of C Ben Miled et al., (2022) [19]. In our study, there was no significant difference in total NE dosing, duration of NE infusion, or duration of ICU stay between the two groups, which is in line with the results of the study by C Ben Miled et al., (2022) [19], which revealed no significant difference in the mean norepinephrine infusion rate (*p* = 0.497), duration of norepinephrine infusion, or duration of ICU stay between the two groups. Additionally, the study conducted by QianyiPeng et al., (2024) [20] reported no significant difference in total norepinephrine dosing (*p* value 0.848) or the length of ICU stay (*p* value 0.062). Regarding total NE dosing calculation in our clinical trial, we used NE tartrate salt with salt to base ratio of 2 [23] (each ampoule of NE contains 8 mg NE tartrate that is equivalent to 4 mg NE base) diluted to achieve 80 µg NE in each ml. The chemical structure of the salt formula ensures drug stability and solubility, whereas the base is pure NE, also the NE base is not commercially available  [24]. The outer label of drug packages usually reports the dosage of NE as a salt not base, which may lead to therapeutic errors with NE prescription  [25]. Our study revealed no significant difference in the final SOFA score (*p* value 0.225) or serum lactate level (*p* value 0.914) at the end of the ICU stay. These results were comparable to those of studies by C Ben Miled et al., (2022) [19] which reported no difference in the final SOFA score or lactate level between the two groups, and Qianyi (2024) [20], who reported no significant difference between the two groups in the final SOFA score (*p* value 0.631) or serum lactate level (*p* value 0.445) at the end of the study. Finally, owing to the high variability in the optimal MA*P* and CP*P* among SIE patients and within the same patient during the course of sepsis, we stress the value of personalized, continuous cerebral perfusion monitoring in guiding resuscitation and hemodynamic management of SIE patients to ensure adequate CP*P* and improve prognosis.

### The current study has some limitations

It is a single-center trial of 112 patients, and further multicentric trials with larger sample sizes may be needed to generalize the results.

## Conclusions

Individualizing hemodynamic management by guiding norepinephrine titration to achieve the optimal mean arterial pressure and cerebral perfusion pressure via the transcranial Doppler pulsatility index wasn`t not associated with significant mortality reduction. However, it was associated with a reduction in episodes of cerebral hypoperfusion and an improvement of GCS score among sepsis-induced encephalopathy patients.

## Supplementary Information

Supplementary Material 1

## Data Availability

The datasets analyzed during the current study are available from the corresponding author on reasonable request.

## Abbreviations

SSC: Surviving sepsis campaign
SIE: Sepsis-induced encephalopathy
TCD: Transcranial Doppler
PI: Pulsatility index
MAP: Mean arterial pressure
CPP: Cerebral perfusion pressure
NE: Norepinephrine
SOFA: Sequential organ failure assessment
ICU: Intensive care unit
GCS: Glasgow coma scale
CBF: Cerebral blood flow
ICA: Internal carotid artery
CCA: Common carotid artery
PSV: Peak systolic velocity
EDV: End diastolic velocity
MFV: Mean flow velocity
MCA: Middle cerebral artery
PW: Pulsed wave
BMI: Body mass index
UTI: Urinary tract infection
