# Supplementary material for: Norepinephrine titration in patients with sepsis-induced encephalopathy: cerebral pulsatility index compared to mean arterial pressure guided protocol: randomized controlled trial
Source: BMC Anesthesiol. 2025 Jan 4;25:5. doi: 10.1186/s12871-024-02814-0 (PMC11699758; doi:10.1186/s12871-024-02814-0)
Supplement: Supplementary file 1 — Supplementary Material 1 [file 12871_2024_2814_MOESM1_ESM.pdf]

EGYTRANSCRIPT
LANGUAGE SOLUTIONS

ENGLISH REVIEW CERTIFICATE

THIS IS TO CERTIFY THAT EGYTRANSCRIPT HAS PROVIDED ENGLISH EDITING TO THE PAPER TITLED "NOREPINEPHRINE TITRATION IN PATIENTS WITH SEPSIS-INDUCED ENCEPHALOPATHY: CEREBRAL PULSATILITY INDEX COMPARED TO MEAN ARTERIAL PRESSURE GUIDED PROTOCOL: RANDOMIZED CONTROLLED TRIAL."

AUTHORS: MAI SALAH SALEM

TO: MAI SALAH SALEM

Authorized by Mohamed Mourad

EGY Transcript Language Solutions

16 Iran St.
Dokki
Giza
Egypt
www.egytranscript.com

EGY Transcript Language Solutions
